# Systemic Acrolein Elevations in Mice With Experimental Autoimmune Encephalomyelitis and Patients With Multiple Sclerosis

**DOI:** 10.3389/fneur.2018.00420

**Published:** 2018-06-15

**Authors:** Melissa Tully, Jonathan Tang, Lingxing Zheng, Glen Acosta, Ran Tian, Lee Hayward, Nicholas Race, David Mattson, Riyi Shi

**Affiliations:** ^1^Weldon School of Biomedical Engineering, Purdue University, West Lafayette, IN, United States; ^2^Medical Scientist Training Program, Indiana University School of Medicine, Indianapolis, IN, United States; ^3^Department of Basic Medical Sciences, Purdue University, West Lafayette, IN, United States; ^4^Department of Neurology, Indiana University School of Medicine, Indianapolis, IN, United States

**Keywords:** oxidative stress, 3-HPMA, aldehyde, inflammation, lipid peroxidation

## Abstract

Demyelination and axonal injury are the key pathological processes in multiple sclerosis (MS), driven by inflammation and oxidative stress. Acrolein, a byproduct and instigator of oxidative stress, has been demonstrated as a neurotoxin in experimental autoimmune encephalomyelitis (EAE), an animal model of MS. However, due to the invasive nature of acrolein detection using immunoblotting techniques, the investigation of acrolein in MS has been limited to animal models. Recently, detection of a specific acrolein-glutathione metabolite, 3-HPMA, has been demonstrated in urine, enabling the noninvasive quantification of acrolein for the first time in humans with neurological disorders. In this study, we have demonstrated similar elevated levels of acrolein in both urine (3-HPMA) and in spinal cord tissue (acrolein-lysine adduct) in mice with EAE, which can be reduced through systemic application of acrolein scavenger hydralazine. Furthermore, using this approach we have demonstrated an increase of 3-HPMA in both the urine and serum of MS patients relative to controls. It is expected that this noninvasive acrolein detection could facilitate the investigation of the role of acrolein in the pathology of MS in human. It may also be used to monitor putative therapies aimed at suppressing acrolein levels, reducing severity of symptoms, and slowing progression as previously demonstrated in animal studies.

## Introduction

Multiple sclerosis (MS) is a presumed autoimmune demyelinating central nervous system disease that affects approximately 2.5 million people worldwide (1). To date, the etiology of MS remains incompletely characterized and, as a result, therapeutic approaches are limited and highly reliant upon suppressing or modulating the overactive immune system to alleviate symptoms and decelerate disease progression (1, 2). A growing number of studies have implicated reactive oxygen species as key mediators of pathological processes associated with the disease, including demyelination and axonal injury (3, 4). However, application of free radical scavengers to curtail oxidative stress conferred limited and somewhat variable symptomatic improvement in the experimental autoimmune encephalomyelitis (EAE) model, prompting investigation of alternative compounds associated with oxidative stress for pharmacological targeting (1–3).

Acrolein, 2-propenal, produced by way of reactive oxygen species instigated lipid peroxidation, has emerged as a potent pro-inflammatory neurotoxin, capable of reacting with lipid, protein, and DNA and perpetuating inflammation and oxidative stress through a feed-forward mechanism (5–11). Recently, acrolein has been shown to be elevated significantly in EAE, an animal model of MS (12), and to inflict damage to myelin, axolemma, and mitochondria both directly and indirectly (13–18). Furthermore, attenuation of acrolein through treatment of EAE with hydralazine, an FDA-approved anti-hypertensive that is known to be an acrolein scavenger (16, 17, 19–23), resulted in a reduction of central nervous system (CNS) acrolein levels which correlated with improved behavioral outcomes and delayed symptomatic onset in EAE mice (12). These data suggest a pathogenic role of acrolein in EAE. It is thus important to determine whether acrolein has a similar role in the pathogenesis of MS. However, clinical determination of acrolein levels *in vivo* in MS patients has not been conducted due to technical difficulties.

Due to recent advances in acrolein detection techniques, minimally invasive quantification of systemic acrolein levels can be achieved through the measurement of 3-hydroxypropyl mercapturic acid (3-HPMA), a specific acrolein-glutathione metabolite, in urine using LC/MS/MS (24–27). Recently, the 3-HPMA quantification method has been successfully applied in an animal model of spinal cord injury (27). In contrast to previous methods utilized exclusively in animal studies, such as immunoblotting which required animal euthanasia to harvest fresh CNS tissue (12, 16, 28–32), this approach allows for the assessment of acrolein levels without the need of sacrificing the animal (27, 33), and thus permits the translation of the 3-HPMA measurement technique to clinical scenarios.

In the current study, using this non-invasive technique, we have found that 3-HPMA levels are significantly elevated in both urine and serum in MS patients compared to healthy individuals. This indicates that 3-HPMA measurement is a feasible, effective assessment of systemic acrolein level in MS patients. The evidence of elevated acrolein in MS in both human patients and animal models is consistent with the proposed notion that acrolein is involved in the pathogenesis of MS. Considering the demonstrated toxicities of acrolein in myelin and axons, we speculate that acrolein is likely a therapeutic target in MS. Furthermore, this method has also made it possible for acrolein to serve as a biomolecule that could potentially be noninvasively monitored to aid in diagnosis, predict disease course, and guide treatment regimens.

## Materials and methods

### Animal preparation

Rodent studies were conducted in accordance with guidelines mandated by the Purdue Animal Care and Use Committee at Purdue University, West Lafayette, IN, USA. The current study was specifically approved by the Purdue Animal Care and Use Committee. Eight-week-old C57BL/6 female mice (Harlan Laboratories, Indianapolis, IN, USA) were maintained in laboratory animal housing facilities for 2 weeks prior to EAE induction to minimize potential effects of stress.

#### EAE model induction and behavioral assessment

Ten-week-old mice were injected subcutaneously over rostral and caudal ends of the spinal column with 0.1 mL MOG35-55/CFA emulsion (EK-0115, Hooke Laboratories, Lawrence, MA, USA). A 0.1 mL volume of deconjugated pertussis toxin (EK-0115, Hooke Laboratories) was administered intraperitoneally on the day of MOG application and again 22–26 h later to aid in permeabilizing the blood brain barrier. A widely utilized 5-point behavioral scoring system (12, 34) was employed to assess motor function by monitoring signs of paralysis and observing gait when animals were placed on a metal grate. Mice were scored in accordance with the following scale: 0, no deficit; 1, limp tail only; 2, hind limb paresis, without leg dragging; 3, partial hind limb weakness with one or both legs dragging; 4, complete hind limb paralysis; 5, moribund, paralysis in hind limbs and possibly in one forelimb. The behavioral score and weight of animals were determined and recorded daily for the duration of the study.

Irreversible dehydration or 20% loss of body weight were used as criteria for humane endpoints requiring euthanasia prior to experimental endpoints. No animals died prior to the experimental endpoint in this study. Ketoprofen (5 mg/kg, subcutaneous) was used to alleviate distress or suffering indicated by quiet posture, decreased activity, self-mutilation, or abnormal respiratory patterns.

#### Dot immunoblotting

Spinal cords were harvested from mice following exsanguination and perfusion of oxygenated Kreb's solution as described in prior publications (12). The procedure of exsanguination was performed under anesthesia with a ketamine (90 mg/kg)-xylazine (10 mg/kg) mixture through intraperitoneal injection. The anesthetized mice were then perfused transcardially with a cold (15°C) oxygenated Krebs solution to remove the blood and lower core temperature. The fresh tissues were incubated with 1% Triton solution and Protease Inhibitor Cocktails, (Sigma-Aldrich, Product #: P8340) homogenized (Kontes Glass Co.) and incubated on ice for at least 1 h. Samples were then centrifuged at 13,500 g and 4°C for a minimum of 30 min.

BCA protein assay was performed to ensure equal loading for all samples. Samples were transferred to a nitrocellulose membrane using a Bio-Dot SF Microfiltration Apparatus (Bio-Rad, Hercules, CA, USA). The membrane was blocked for 1 h in blocking buffer (0.2% Casein and 0.1% Tween 20 in PBS) and transferred to a solution where polyclonal rabbit anti-acrolein antibody (Novus Biologicals) was dissolved, with a ratio of 1:1,000, in blocking buffer with 2% goat serum and 0.025% sodium azide, for 18 h at 4°C. The membrane was then washed blocking buffer and incubated for 1 h in a solution of 1:10,000 alkaline phosphatase conjugated goat anti-rabbit IgG (VECTASTAIN ABC-AmP Kit). Final washes of the blocking buffer followed by 0.1% Tween 20 in Tris-buffered saline were performed before the membrane was exposed to substrate of the ABC-AMP kit and visualized by chemiluminescence. Band density was quantified using Image J (NIH) and expressed as arbitrary unit.

#### Animal urine collection

Mice were housed in metabolic cages, designed to obtain urine samples, for 12–24 h. Regular food and water were supplied during the sample collection period. Samples of approximately 500 μL were obtained from each animal at peak behavioral deficit between days 21 and 23. Samples were then transferred to 1 mL centrifuge tubes and frozen at −80°C until biochemical analyses were performed.

#### Hydralazine application

Hydralazine hydrochloride (Sigma, St. Louis, MO, USA) was dissolved in phosphate buffered saline, sterilized through a filter, and subsequently stored at 4°C. Hydralazine at a dosage of 1 mg/kg (Body Weight) was applied daily through intraperitoneal injections (IP), commencing from the day of induction until the end of study period (22 days post induction).

### Human subject enrollment

All human specimens were collected at the Indiana University Multiple Sclerosis Center, Department of Neurology, Indiana University School of Medicine, Indianapolis, IN, USA. Criteria for subject selection consisted of an MS diagnosis and that the patient not be receiving corticosteroids at the time of the sample collection. In this regard, it is important to note that many patients were on various FDA-approved MS immunotherapies at the time of sample collection. This study was carried out in accordance with guidelines set forth in the protocol approved by the Indiana University Human Subjects Institutional Review Board. All patients provided written informed consent in accordance with the Declaration of Helsinki using a consent form approved by the Institutional Review Board.

#### Clinical urine collection

Subjects were provided with a specimen cup, without preservative, for urine sample collection. Urine samples were then pipetted into labeled cyrovials and immediately stored at −80°C prior to being transported to Purdue University on dry ice. Upon arrival, samples were immediately stored at −80°C until analysis.

#### Clinical serum collection

Venous blood samples were obtained by standard technique (BD Vacutainer® Safety-Lok™ Blood Collection Set 23, Gauge 3/4 Inch Safety Needle, 12 Inch Tubing Sterile) and directly placed into a BD Vacutainer® Plus Venous Blood Collection Tube Serum Tube Clot Activator 13 × 100 mm 6 mL BD Hemogard™ Closure Plastic Tube. Following collection, samples were incubated for 15 min at room temperature to facilitate clotting. The samples were then centrifuged at 2,800 rpm for 15 min (Beckman GS-6R) and transferred to a labeled cyrovial and stored at Thermo Scientific −80°C. Samples were then transported to Purdue University on dry ice and stored at −80°C until analysis.

### 3-HPMA quantification using LC/MS/MS and standard preparation

#### 3-HPMA quantification

3-hydroxypropyl mercapturic acid (3-HPMA) was quantified in urine according to Eckert et al. (35). Solid phase extraction with Isolute ENV+ cartridges (Biotage, Charlotte, NC) was used to prepare each sample before LC/MS/MS analysis. Cartridges were conditioned with 1mL of methanol, 1mL of water, and 1mL of 0.1% formic acid in water in succession. Urine or serum sample aliquots of 500 μL were combined with 200 ng of deuterated 3-HPMA (d3-3-HPMA) (Toronto Research Chemicals Inc., New York, Ontario), 500 μL of 50 mM ammonium formate and 10 μL of undiluted formic acid and pipetted into the prepared ENV+ cartridges. The cartridges were then washed twice with 1 mL of 0.1% formic acid and 1 mL of 10% methanol/90% 0.1% formic acid in water in succession. The cartridges were dried with nitrogen gas and subsequently eluted with three volumes of 600 μL methanol + 2% formic acid which were combined and dried in a rotary evaporation device. Samples were reconstituted in 100 μL of 0.1% formic acid prior to LC/MS/MS analysis.

Quantification of 3-HPMA in the samples was determined using an Agilent 1200 Rapid Resolution liquid chromatography (LC) system coupled to an Agilent 6460 series QQQ mass spectrometer (MS) and a Waters Atlantis T3 2.1mm x 150mm, 3 μm column for LC separation. Water + 0.1% formic acid and acetonitrile + 0.1% formic acid were used as buffers. The peak retention time of 3-HPMA/d3-3-HPMA was 6.8 min. Multiple reaction monitoring was used for MS analysis. A more detailed procedure is outlined in previous publication (27).

#### Creatinine assay

Creatinine quantification was performed to provide an internal standard normalize urine 3-HPMA measurements. Sample creatinine concentrations were determined through the use of a urinary creatinine assay kit (Cayman Chemical Company, Item No. 500701). Urine samples were diluted for 12 × and 24 × prior to measurement and alkaline picrate solution was prepared following the procedure delineated in the assay manual. The diluted samples and creatinine standards were then loaded into a 96 well plate and incubated with the alkaline picrate solution at room temperature for 20 min. Absorbance at 490–500 nm was determined using a standard spectrophotometer and the results were recorded as the initial reading. Following the initial reading, 5 ul of acid solution was added to each sample and incubated on a shaker at room temperature for an additional 20 min. A spectrophotometer (absorbance at 490–500 nm) was used again to determine the final reading following addition of the acid. The differences between the initial and final absorbance measurements were used for quantitative analysis.

BSA assessment was used as a normalization factor for the serum 3-HPMA measurements. Protein concentrations using bovine serum albumin were quantified using the Bicinchoninic Acid protein assay kit (Pierce, Rockford, IL, USA). Serum samples were prepared in a 1:100 dilution and loaded into a 96 well plate along with BSA standards in triplicates. BSA reagent was then added to all wells and the samples were incubated at 37 C for 30 min. Following incubation the absorbance of the samples at 560–570 nm was determined using SPECTRAMAX (Molecular Devices, Sunnyvale, CA, USA).

### Statistical analysis

Student's *t*-test (unpaired, two-tailed) was used to compare various acrolein measurements between control and EAE/MS groups in different conditions. For comparisons involving three or more groups, ANOVA and *post-hoc* Newman–Keul's test were used to compare the data. Linear correlation was expressed by Pearson correlation coefficient (r). Results are expressed as mean ± SEM. *P* < 0.05 was considered statistically significant.

## Results

### CNS and systemic elevation of acrolein in EAE mice

**Figure 1:**
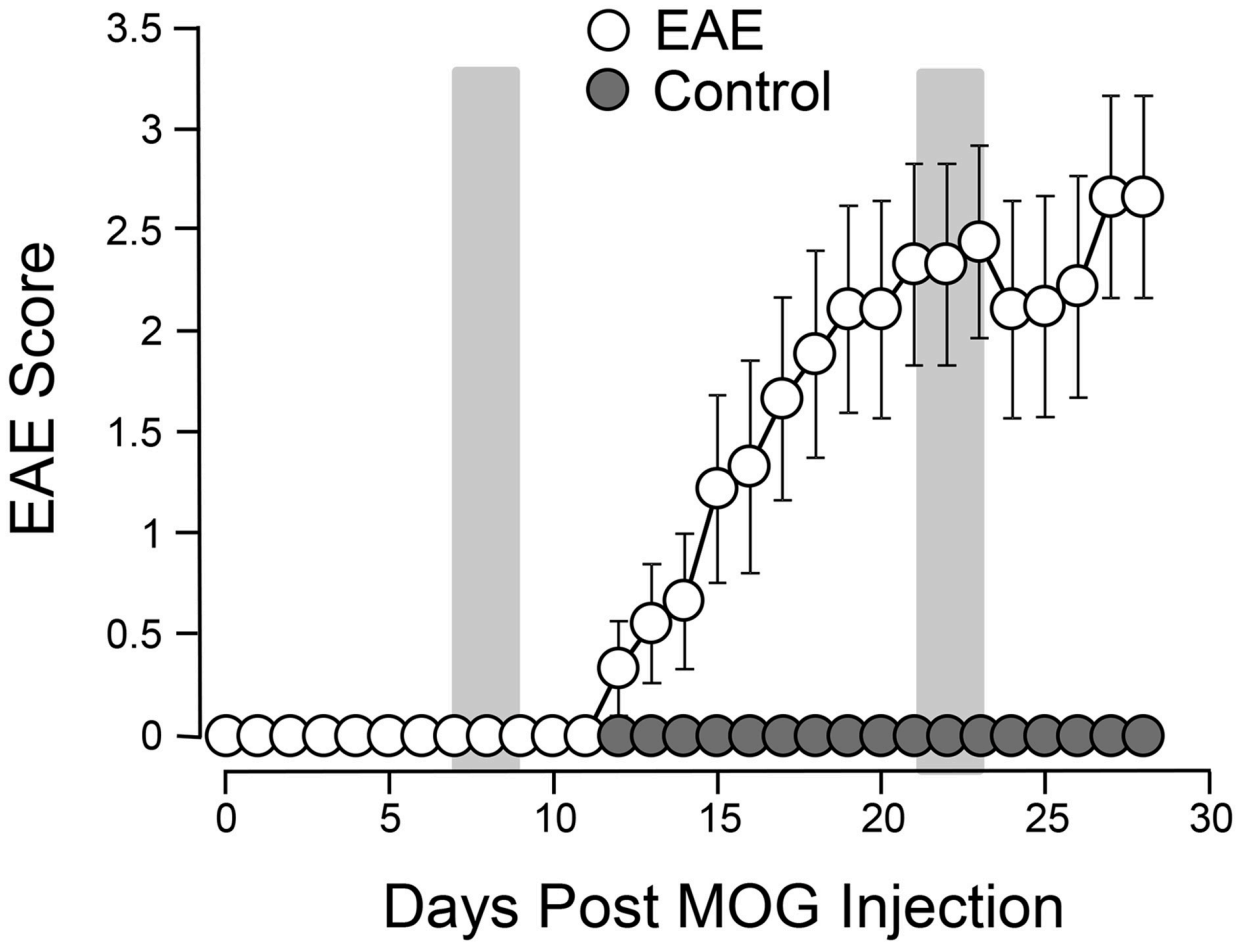
Behavioral deficits in EAE mice following induction. A total of 9 mice were subjected to MOG injection. The same number of age matched uninured mice were used to serve as controls. The motor deficits typical of EAE were scored daily for 4 weeks. The motor function of control mice was also observed for the same period of time for comparisons. The mean value (and SEM) of both control and EAE mice are plotted against time post induction. The two shaded areas indicate the time periods (7–9 days) and (21–23 days) when urine 3-HPMA samples were collected and assessed using LS/MS/MS. Acrolein-lysine adduct levels in spinal cord tissue were quantified using dot blot. Both urine 3-HPMA and tissue acrolein-lysine adduct were used to assess acrolein levels. Note the steady rises of EAE score beginning at around 11–12 days and peak attained around 21–22 days post induction. Data expressed as mean ± SEM.

**Figure 2:**
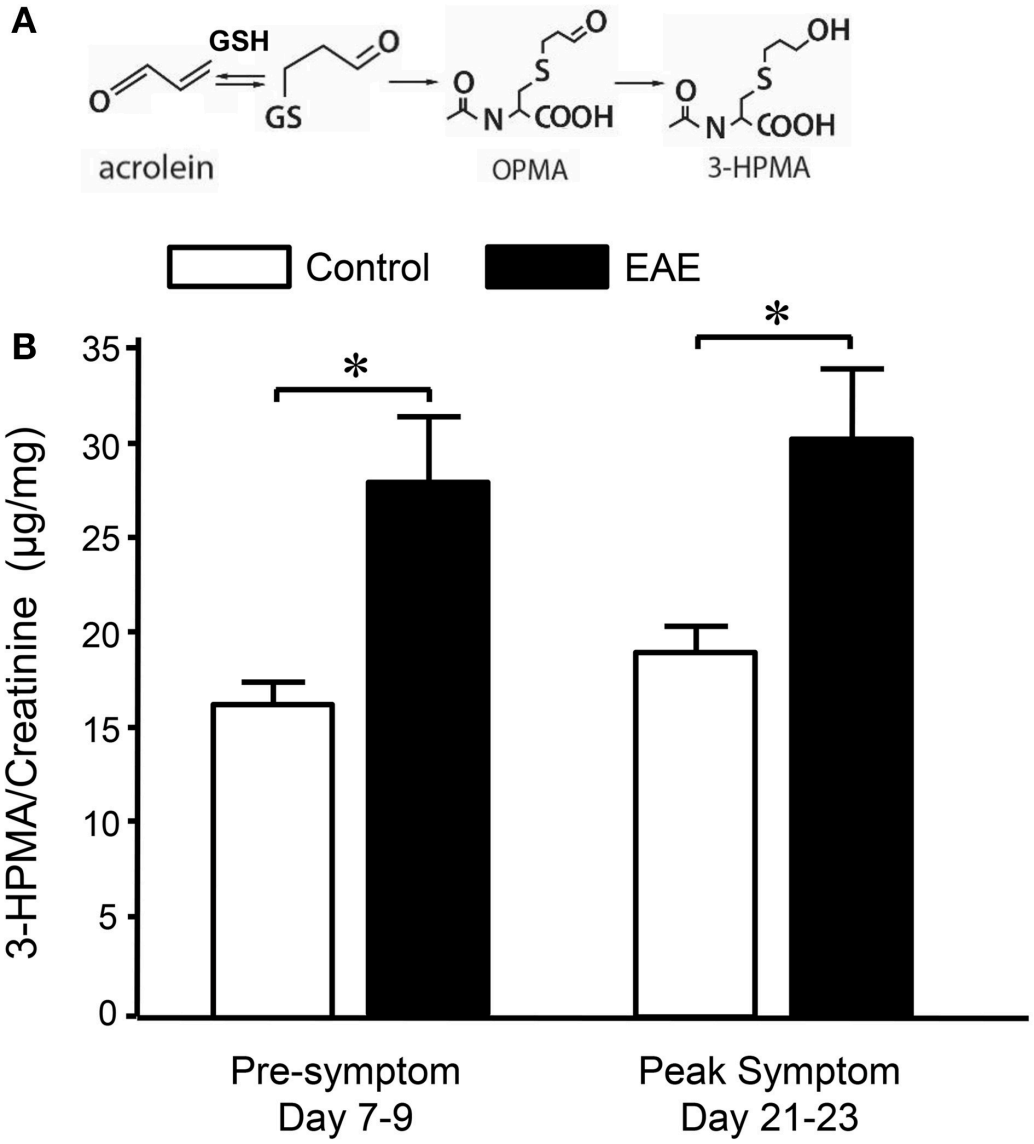
Determination of acrolein concentration through urine 3-HPMA measurement in control and EAE mice during pre or peak symptomatic stage. **(A)** Chemical reaction of acrolein with glutathione (GSH) and production of subsequent metabolites OPMA and 3-HPMA. **(B)** Bar graph depicts the ratio of 3-HPMA and creatinine measured in urine of control and EAE mouse. Urine samples were collected approximately 7–9 days and 21–23 days after MOG injection in EAE mouse which correspond to pre and peak symptomatic stage (see also Figure 1). Urine samples were also collected from age matched control mice at similar time periods. Each urine sample represents an accumulative volume of a 24 h period. Note the increase of 3-HPMA in urine in EAE mice in both pre and peak symptomatic periods when compared to control group (^*^P < 0.05 when compared to control, ANOVA and *post-hoc* Newman-Keul's test). *N* = 9 in each group of 3-HPMA measurement in urine. Data expressed as mean ± SEM.

**Figure 3:**
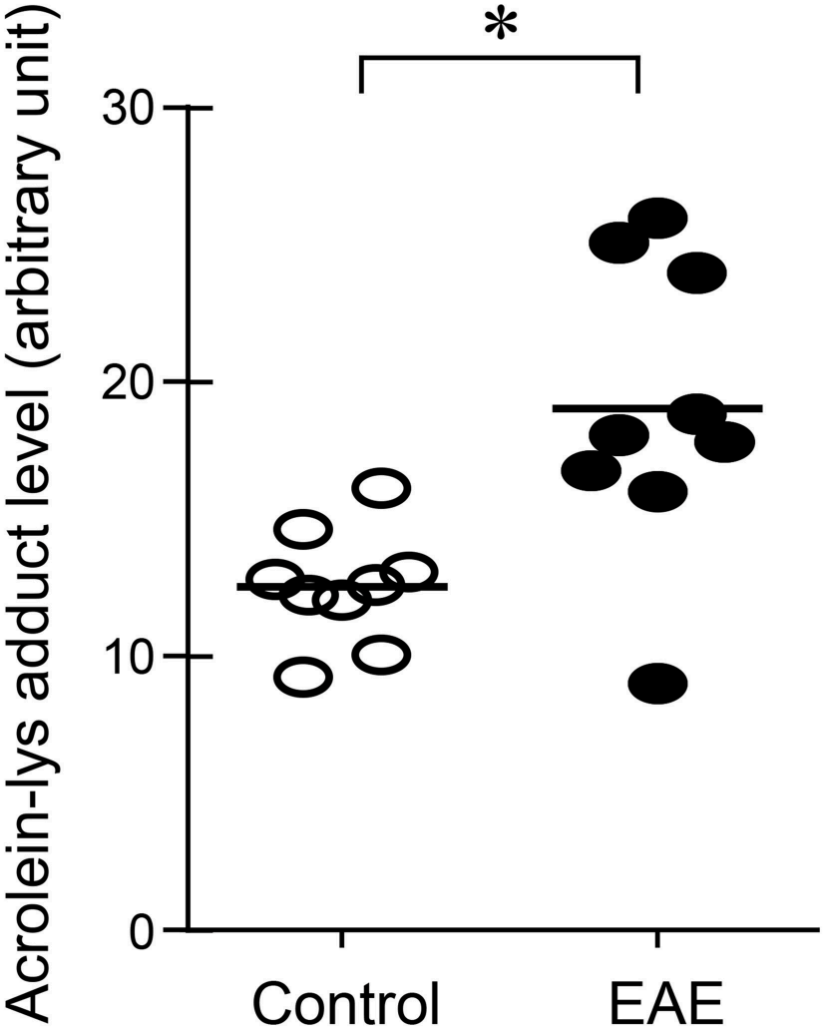
Determination of acrolein changes in spinal cord tissue by immunoblotting in EAE mice. The acrolein-lysine adducts in control and in EAE were detected using Bio-Dot SF microfiltration apparatus. Band intensities were analyzed using image J (NIH) and expressed in arbitrary units. Note the scatter plot demonstrated an increase of acrolein-lysine adducts in EAE. (^*^P < 0.01 when compared to control, *t*-test). *N* = 9 in each group of acrolein measurement in tissue.

**Figure 4:**
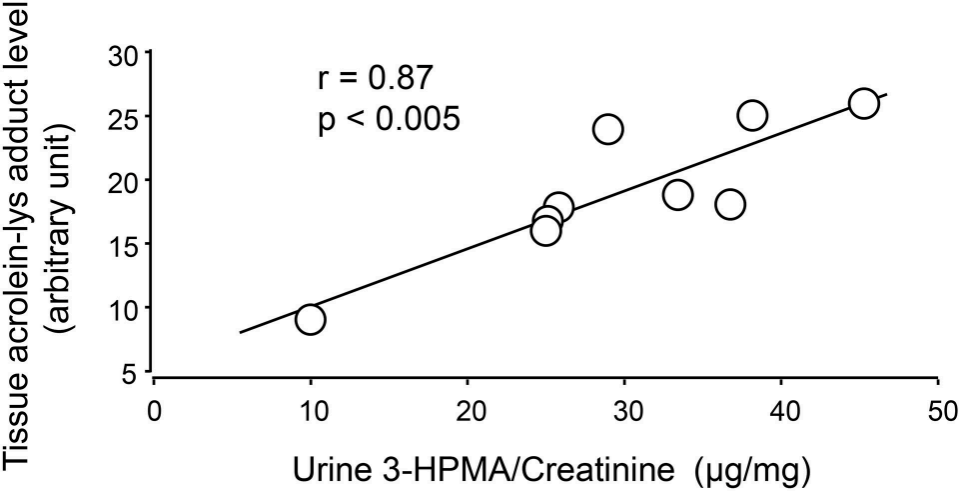
Correlation of 3-HPMA levels in urine and acrolein-lysine adducts in spinal cord tissue of EAE mice. The urine 3-HPMA is plotted against tissue acrolein-lysine adducts for 9 EAE mice showing the relationship between these two parameters. As indicated, the increase of urine 3-HPMA correlated with the elevation of tissue acrolein-lysine adducts. Statistical analysis of correlation revealed a Pearson correlation coefficient (*r*) value of 0.87 (*p* < 0.005, two tailed).

**Figure 5:**
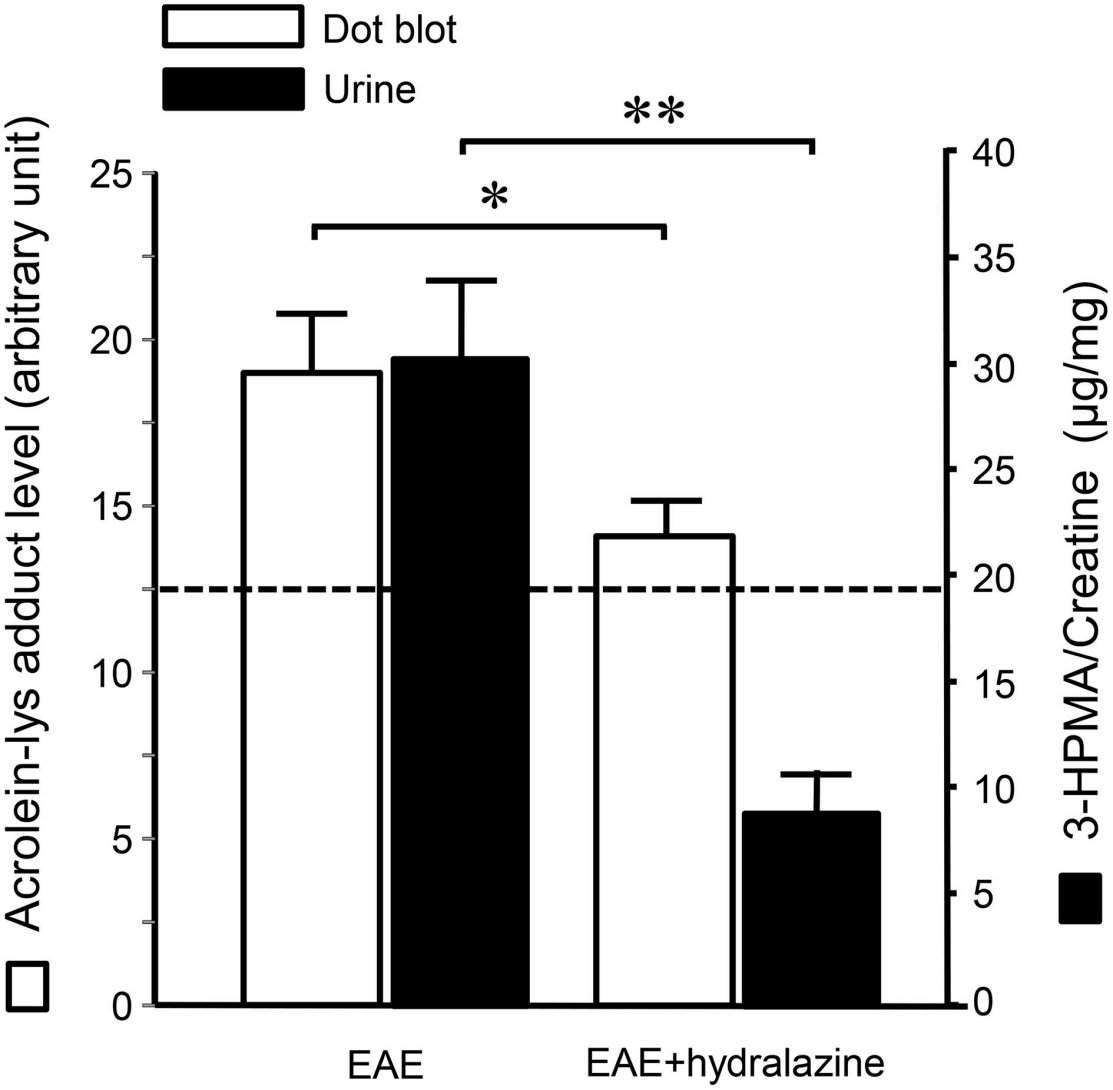
Hydralazine effectively suppressed acrolein-lysine adducts in spinal cord and 3-HPMA in urine in EAE mice. In EAE plus hydralazine treatment group, hydralazine (1 mg/kg) was administered IP daily starting immediately following induction. Urine samples in both groups were collected 21–23 days post induction when the behavior deficits peak. Dotted line represent the value from age matched mice served as controls. Note the suppression of either acrolein-lysine adducts in spinal cord tissue, or 3-HPMA in urine, in EAE plus hydralazine group compared to EAE group (^*^P < 0.05; ^**^P < 0.01, ANOVA and *post-hoc* Newman-Keul's test). *N* = 9 in each group of acrolein measurement in tissue and 3-HPMA measurement in urine.

Systemic acrolein levels based on urine samples in EAE mouse were determined through the quantification of 3-HPMA using LC/MS/MS (27). Urine was collected from controls and EAE mice during pre-symptomatic period of days 7–9, and when peak deficit occurred at days 21–23 post induction (Figure 1). At days 7–9, EAE mice displayed an elevated level of 3-HPMA (27.3 ± 4.2 μg/mg creatinine) which is significantly higher than control mice (16.9 ± 0.8 μg/mg creatinine, *p* < 0.05). Similarly, EAE mice demonstrated significantly elevated urine 3-HPMA levels (30.5 ± 3.6 μg/mg creatinine) relative to their healthy counterparts (19.5 ± 1.0 μg/mg creatinine, *p* < 0.05) (Figure 2) at days 21–23 post induction. In addition to urine acrolein levels, we also quantified the correspondent acrolein level in spinal cord tissue using dot immunoblotting. Specifically, both EAE and control groups were sacrificed at day 28 post induction and spinal cord tissue was harvested to assess local acrolein concentrations within the CNS using a dot immunoblotting assay. We have found that EAE mice exhibited significantly elevated intrinsic levels of acrolein-lysine adducts (19.1 ± 1.8 au) relative to the control group (12.5 ± 0.7 au, *p* < 0.01) (Figure 3). A correlation analysis between urine 3-HPMA and spinal cord tissue acrolein-lysine adducts in EAE mice at the peak symptomatic stage has revealed a significant positive relationship with a Pearson correlation coefficient (*r*) value of 0.87 (*p* < 0.005) (Figure 4). In a separate group, EAE mice received daily IP injection of hydralazine treatment (1 mg/kg), starting the day of induction, and the urine was collected at 21–23 post induction, and the spinal cord tissue were collected 28 days post induction and treatment. As indicated in Figure 5, the value of urine 3-HPMA in EAE plus hydralazine group (9.25 ± 1.54 μg/mg creatinine) is significantly less than that in EAE group when examined 21–23 days post induction (29.9 ± 3.6 μg/mg creatinine, *P* < 0.01). Similar, the value of acrolein-lysine adducts in spinal cord tissue (14.2 ± 1.1 au) is also significantly less than that in EAE group (19.1 ± 1.8 au, *P* < 0.05).

### Multiple sclerosis patients exhibited increased 3-HPMA in urine and serum

**Figure 6:**
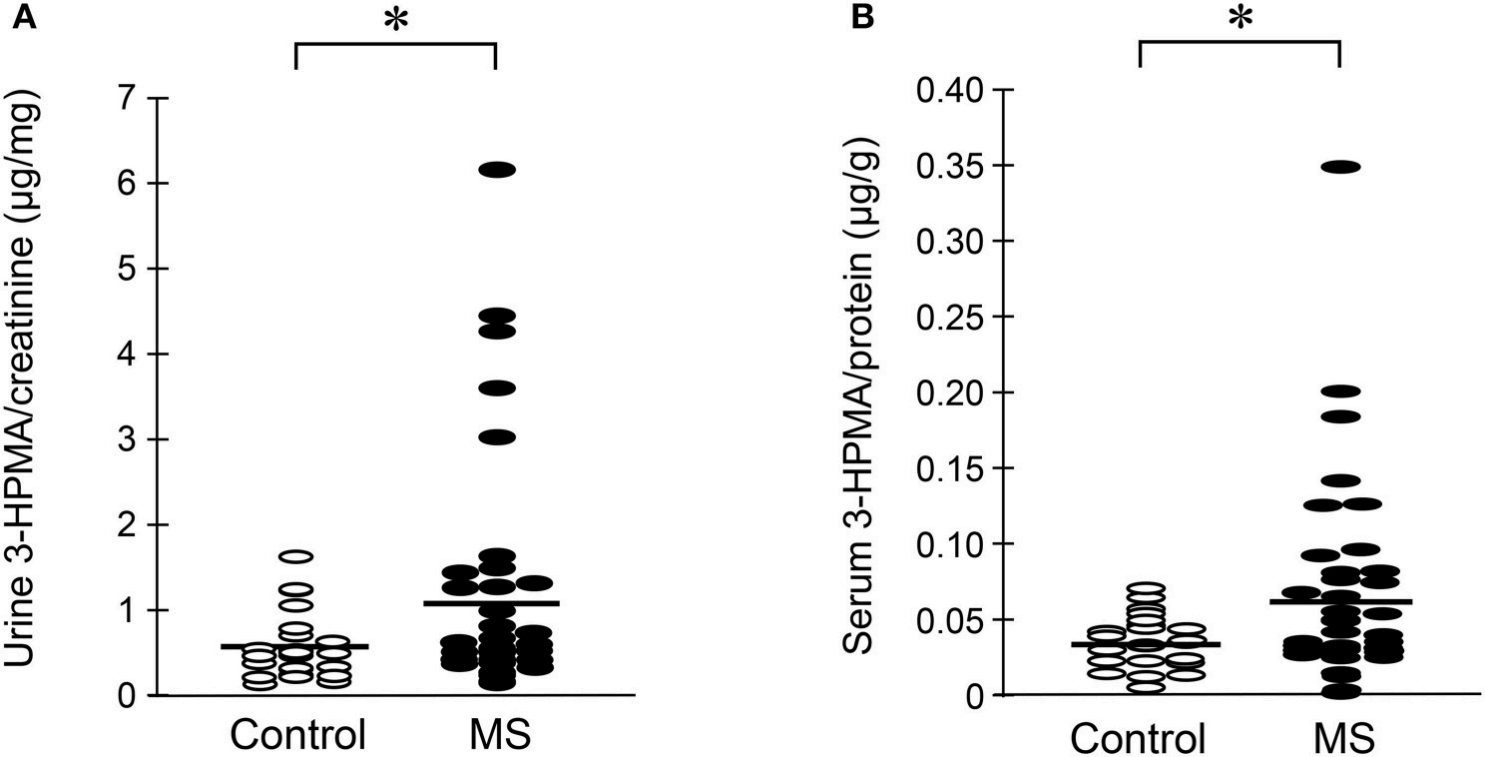
Determination of acrolein concentration through urine and serum 3-HPMA measurement in MS patients and healthy individuals. The MS patient group including 31 relapsing-remitting (RRMS), 8 secondary progressive (SPMS), and 1 primary progressive (PPMS) types of MS. **(A)** A scatter plot including all the data points to reveal the range and distribution of measured values of urine 3-HPMA. Solid lines indicate mean 3-HPMA in both MS and healthy control individuals. Note that while many data points of MS patients were distributed in the same range as that of control, there were still multiple points of MS which were greater than that in control, some by multiple folds. Specifically, the mean concentration of 3-HPMA is 1.094 ± 0.212 μg/mg creatinine for MS patients (*N* = 40) and 0.570 ± 0.082 μg/mg creatinine for healthy individuals (*N* = 23). Note the increase of 3-HPMA in urine in MS patients. (^*^: *P* < 0.05, *t*-test). **(B)** A scatter plot including all the data points to reveal the range and distribution of measured values of serum 3-HPMA. Solid lines indicate the mean 3-HPMA level in both MS patients and control individuals. Note that while many data points of MS patients were distributed in the same range as that of controls, there were still multiple points of MS that were greater than that of control, some by multiple folds. The mean concentration of 3-HPMA is 0.065 ± 0.009 μg/g protein for MS patients (*N* = 40) and 0.036 ± 0.004 μg/g protein for healthy individuals (*N* = 23). Note the increase of 3-HPMA in serum among MS patients. (^*^: *P* < 0.05, *t-*test).

**Figure 7:**
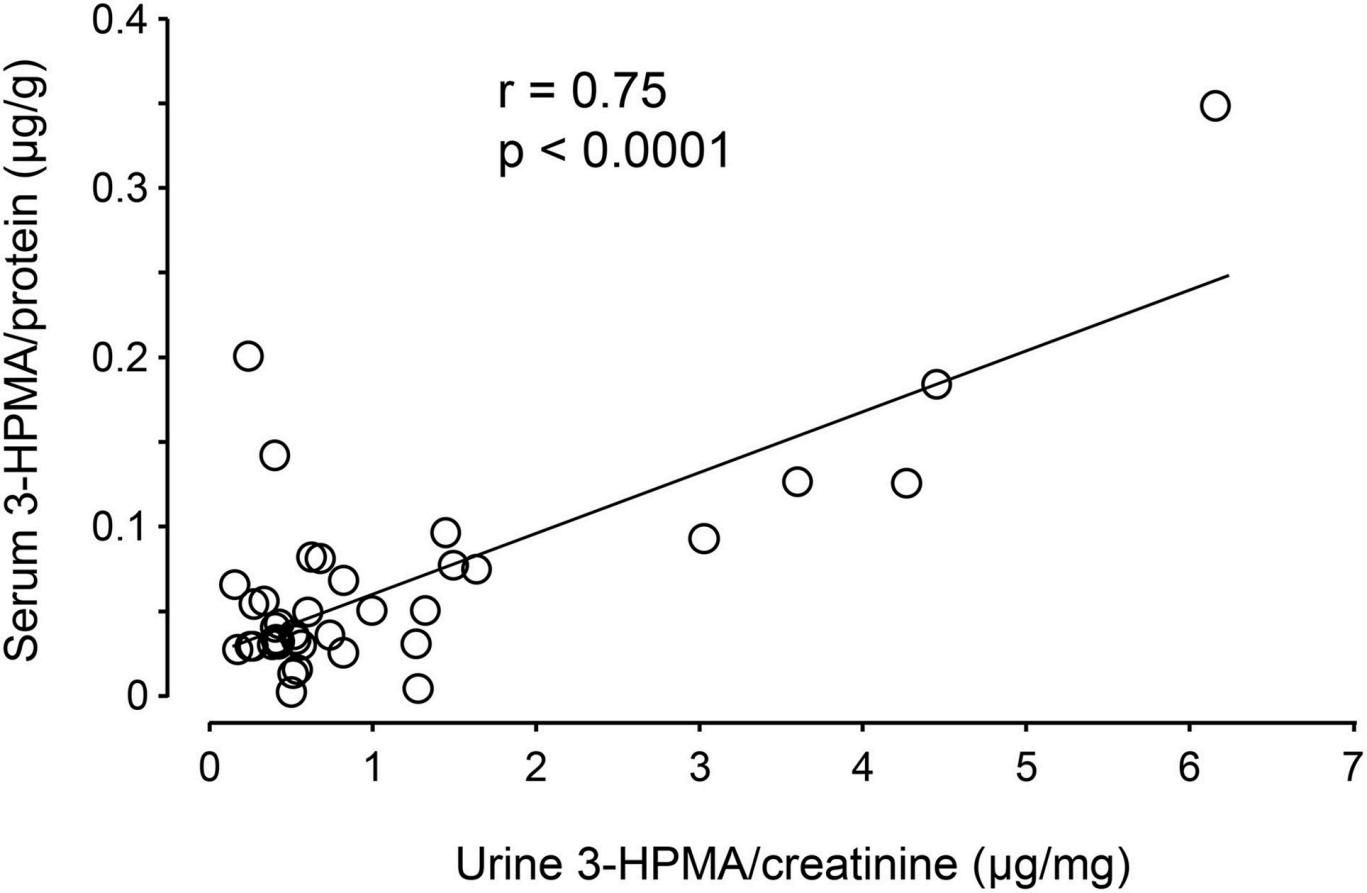
Correlation of 3-HPMA levels in urine and serum in MS patients. The urine 3-HPMA is plotted against serum 3-HPMA for 39 MS patients showing the relationship between these two parameters. Urine and serum were collected at the same time for all patients. As indicated, the increase of urine 3-HPMA seems to be correlated with the elevation of serum 3-HPMA. Statistical analysis of correlation revealed a Pearson correlation coefficient (*r*) value of 0.75 (*p* < 0.0001, two tailed).

Urine and serum samples were collected from diagnosed MS patients (*n* = 40) and volunteer healthy controls consisting of mainly office staff and family members of patients (*n* = 23). Among MS patients, there were 27 females and 13 males, while in the control group, there were 18 females and 5 males. The average age at the time of specimen collection was 45.9 years for MS patients and 47.7 years for control individuals. Among MS patients, there were 31 who were diagnosed as relapsing-remitting MS (RRMS), 8 were diagnosed as secondary progressive MS (SPMS), and 1 as primary progressive MS (PPMS). There were 38 patients who were diagnosed as not in the relapsing stage and 2 (one as SPMS and another as RRMS) were in relapse at the time of specimen collection. Only non-smokers in both MS and control groups were included in this study. Acrolein content within the specimens (both urine and serum) was estimated through the assessment of 3-HPMA using LC/MS/MS. Figure 6A depicts 3-HPMA measurements using scatter plot. Mean 3-HPMA levels detected in the urine of MS patients (1.094 ± 0.212 μg/mg creatinine) were significantly elevated relative to healthy controls (0.570 ± 0.082 μg/mg creatinine, *p* < 0.05). Results obtained following quantification of 3-HPMA in patient serum specimens corresponded with the data obtained from urine analysis for both the MS group (0.065 ± 0.009 μg/g protein) and the control group (0.036 ± 0.004 μg/g protein), in which MS patients demonstrated a significant elevation compared to control (*p* < 0.05, Figure 6B). Interestingly, a correlation analysis between 3-HPMA measurements in MS patient urine and serum samples, revealed a significant positive relationship with a Pearson correlation coefficient (*r*) value of 0.75 (*p* < 0.0001) (Figure 7).

## Discussion

In the current study, using a non-invasive method, we have shown that 3-HPMA, a stable acrolein metabolite, is significantly elevated in urine in EAE mice at pre-symptomatic stage (7–9 days post induction) and peak symptomatic stage (21–23 days post induction). Such elevation of 3-HPMA in urine is correlated with an increase of acrolein in spinal cord tissue, and both can be reduced with the application of hydralazine (1 mg/kg). Furthermore, we have also revealed that 3-HPMA is significantly elevated in the urine and serum of MS patients compared to healthy controls. To our knowledge, this is the first reported study to non-invasively quantify acrolein, an alleged pathogen in myelin and neuronal damage in MS, in both human and animal models using a 3-HPMA assay. Notably, we also present the first clinical evidence that MS patients exhibit elevated systemic acrolein concentrations.

Based on the established evidence of neurotoxicity of acrolein (5, 6, 13–18) and its elevation in both EAE mice and human MS patients (12), we suggest that acrolein likely plays a critical role in neuronal tissue damage characteristic of MS. Considering the fact that anti-acrolein treatment has been shown to reduce acrolein levels in the current and prior studies, and offer neuroprotection in EAE mice (12), we speculate that acrolein may also serve as a therapeutic target for pharmacological intervention and treatment evaluation for anti-acrolein therapy in a clinical setting. An acrolein-based therapeutic approach may represent a novel strategy in the management of MS, a devastating neurological condition with limited therapeutic approaches for symptom management.

One interesting and unexpected finding of the current study is that urine 3-HPMA is elevated before the emergence of symptoms in EAE mice. We feel this likely represents the augmented level of immunological/inflammatory activity that precedes the motor deficits in this animal model of MS. Consistent with this, it has been shown that significant membrane damages can be detected before the emergence of symptoms in EAE mice (36) while acrolein is known to inflict membrane destruction (13). Similarly, allodynia, a pathology which can stem from inflammation and acrolein (30), can be detected before any signs of neurological deficits in EAE mice (37). These observations are also consistent with the *in vitro* and *ex vivo* data that acrolein-mediated damage is time-dependent, meaning the *in vivo* behavioral deficits likely appear following a continuous acrolein exposure (15, 17). Therefore, the elevation of urine 3-HPMA could be used as a diagnostic and/or monitoring tool to associate and predict the emergence of behavioral impairments such as motor deficits, neuropathic pain, and other common MS symptoms. However, while it is possible that pre-symptomatic neuro-inflammation can be detected in human, cautions needs to be taken when translating such results from animal to human, considering the cause of the disease in human MS and in EAE are obviously different.

The success of non-invasive acrolein detection in urine would not only allow longitudinal animal *in vivo* studies of acrolein dynamics, evaluation of potential anti-acrolein therapies, but also permits the detection of acrolein in human patients (24–27). As such, the clinical component of this study exclusively relied on 3-HPMA quantification as an acrolein detection method. However, 3-HPMA was independently quantified in both urine and serum samples to ensure the reliability of the measurements, and both showed significant and correlative elevation (Figure 7), It is worth noting that, while most of reported detection of 3-HPMA have been conducted in urine, we have observed similar phenomenon in blood in the same group of animal subjects animal, which not only further strengthening the validity of 3-HPMA measurements in both urine and serum, but also facilitate the utility of such method.

One limitation of the current human study is the low number of total participations patients, as well as the uneven distribution of patients in some sub groups which potentially hindered statistical analysis. The comparison between patients subgroups, such as male (13) vs. females (27); RRMS (31) and SPMS (8); remission (38) vs. relapse (2), did not yield significance. A more comprehensive longitudinal study with greater statistical power could potentially reveal how 3-HPMA levels correlate with active structural lesions (such as brain and spinal cord lesions identified using neuroimaging), sex, symptomatic presentations (remitting vs. relapsing stage), symptom emergence and progression (RRMS, SPMS, and PPMS), and further solidify the role of acrolein in pathological processes underlying clinical MS.

Furthermore, acrolein detection could help patient selection for better response, as well as to optimize the dosage regimen for an anti-acrolein treatment. For example, while the average of 3-HPMA among MS patients is significantly higher than control, it is conceivable that some MS patients exhibited 3-HPMA values that are closer to controls (Figure 6). Understandably, patients with higher acrolein level may benefit from anti-acrolein therapy more than those without such feature. Non-invasive acrolein detection also allows for dosage determination of acrolein scavengers to be tailored to a specific patient and to assess the effectiveness of acrolein-scavenging therapy. Taken together, the non-invasive detection of acrolein described in the current study could potentially play a critical role in the treatment of illness that is associated with high level of acrolein.

Although we have detected elevated acrolein in both locally through spinal cord tissue and systemically through urine in our animal studies, there are some differences worth noting which could shed light on the future clinical studies on the differences between MS and other neurological conditions such as spinal cord injury. In the current study of EAE, the degree of increase of acrolein elevation in urine is 1.56 greater than control (days 21–23), which is significantly correlated to the increase of acrolein in spinal cord tissue (1.53 greater than control), indicative that acrolein is elevated proportionally in both CNS tissue and general circulation. This differs from the context of an acute neurological injury, such as SCI, where the elevation of acrolein in spinal cord is 4.5 greater than control while urine 3-HPMA only increased by a factor of 1.8 (27). This may reflect the fact that spinal cord tissue damage in SCI initiated by mechanical trauma, is more severe, and causes an intense acute increase of acrolein locally at the injury site, and not diffusely like CNS damage in MS. This may also explain the lesser degree of elevation of 3-HPMA in urine post-SCI because the acrolein concentration is generated from a single lesion site and significantly diluted upon entering systemic circulation. In contrast, MS displays much more diffusive pathology over longer time course, which may explain the similar degree of elevation of acrolein in both CNS and systemic measurements observed in MS animal models. This also suggests that a systemic determination of acrolein in SCI maybe an underestimation of acrolein in spinal cord tissue, which could be several factors higher than that in observed in urine (27). This is in contrast to what is observed in EAE mice, where the systemic measurement of 3-HPMA in urine is a relatively accurate reflection of acrolein level in spinal cord tissue. One related phenomenon is the degree of suppression of acrolein by hydralazine seems to be more prominent in urine than in spinal cord (Figure 5). This is likely due to the more direct access of systemic circulation to drugs through IP injection. Therefore, the effectiveness of anti-acrolein effect of hydralazine based on urine may be an overestimate of its effect in spinal cord tissue.

In summary, the observation of the elevated 3-HPMA levels in MS patients using non-invasive measures will not only further strengthen the proposed pathological role of acrolein in MS, but also present possibilities for its utility in the diagnosis and treatment assessment of MS. Clinical applicability of acrolein-scavenging is further augmented by the availability of multiple off-label FDA-approved acrolein-scavengers, most notably hydralazine, phenelzine, and dimercaprol [(8, 12, 17, 21–23, 31, 38–42)]. Taken together, the efficacious application of non-invasive 3-HPMA measurements in MS, along with the availability of acrolein-suppressing drugs, has underscored the translational nature of acrolein research potentially leading to significant improvements for both the diagnosis and treatment of MS clinically.

## Author contributions

RS and DM conceived and designed the experiments. MT, JT, LZ, GA, RT, LH, and NR performed the experiments. MT, LZ, GA, JT, NR, and RS analyzed the data. MT, JT, RS, and DM wrote the manuscript. All authors discussed the results and commented on the manuscript, and approved the final version of the manuscript.

### Conflict of interest statement

RS is the co-founder and DM is the scientific advisor of Neuro Vigor, a start-up company with business interests of developing effective therapies for CNS neurodegenerative diseases and trauma. The remaining authors declare that work was carried out in the absence of any financial relationships which could be construed as a conflict of interest.

## Abbreviations

3-HPMA: 3-hydroxypropyl mercapturic acid
CNS: Central nervous system
EAE: Experimental autoimmune encephalomyelitis
MS: Multiple sclerosis
PPMS: Primary progressive multiple sclerosis
RRMS: Relapsing-remitting multiple sclerosis
SPMS: Secondary progressive multiple sclerosis.
